# Cardiorespiratory fitness and plasma metabolites in adults: Sex‐dependent insights from a combined regression and machine learning approach

**DOI:** 10.14814/phy2.71208

**Published:** 2026-11-13

**Authors:** Malin Enarsson, Tessa Schillemans, Hannah L. Brooke, Lars Lind, Johan Ärnlöv

**Affiliations:** ^1^ Division of Family Medicine and Primary Care, Department of Neurobiology, Care Sciences and Society (NVS) Karolinska Institutet Stockholm Sweden; ^2^ Center for Clinical Research Dalarna Falun Sweden; ^3^ Medical Epidemiology, Department of Surgical Sciences Uppsala University Uppsala Sweden; ^4^ Department of Medical Sciences Uppsala University Uppsala Sweden; ^5^ School of Health and Social Studies Dalarna University Falun Sweden

**Keywords:** cardiometabolic health, cardiorespiratory fitness, metabolomics, sex differences

## Abstract

Cardiorespiratory fitness (CRF) is associated with reduced cardiovascular disease risk, but the underlying molecular mechanisms remain poorly understood. Metabolomics offers a promising strategy to elucidate these mechanisms. Our aim was to investigate sex‐dependent associations between CRF and plasma metabolites. This cross‐sectional study included 450 participants (225 females, 225 males) from the Swedish population‐based POEM cohort. CRF (VO_2_peak) was assessed by maximal bicycle ergometer testing. Plasma levels of 790 metabolites were quantified using the Metabolon platform. Associations between VO_2_peak and metabolites were examined using sex‐stratified multivariable linear regression and random forest models, adjusted for cardiovascular risk factors. VO_2_peak was associated with 27 metabolites in females and 24 in males (*p* < 0.000063), of which 4 overlapped between sexes and 10 showed sex‐specific associations. Most associations remained significant after adjustment for cardiovascular risk factors. Significant metabolites substantially improved model fit for VO_2_peak (adjusted *R* ^2^: females 26%–53%; males 27%–37%; *p* < 0.001). Random forest variable importance rankings showed substantial overlap with regression findings. In conclusion, CRF was associated with distinct plasma metabolite profiles in females and males, with most metabolites showing sex‐dependent associations and a smaller subset being shared between the sexes. These findings provide new insight into the biological pathways connecting CRF to cardiometabolic health.

## Graphical abstract

Cardiorespiratory fitness (VO_2_peak) was measured by maximal cycle ergometry in 225 women and 225 men from the Swedish POEM cohort, and 790 plasma metabolites were profiled. Cardiorespiratory fitness was associated with 27 metabolites in women and 24 in men, with only 4 overlapping. Most associations persisted after adjustment for cardiovascular risk factors. These findings indicate sex‐dependent metabolite profiles of cardiorespiratory fitness.

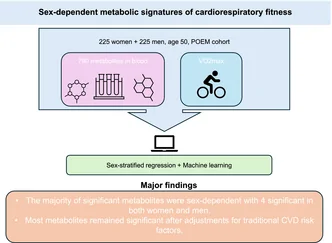

## 1 INTRODUCTION

Cardiorespiratory fitness (CRF) is a potent health marker and is associated with major health benefits including reduced risk of cardiovascular disease (CVD) and all‐cause mortality (Harber et al., 2017; Kodama et al., 2009). Even small increases in CRF have been shown to reduce the risk of CVD and all‐cause mortality by up to 15% (Harber et al., 2017; Kodama et al., 2009). CRF is recognized as a vital sign in clinical practice (Ross et al., 2016). CRF increases with regular physical activity, and CRF is also modulated by genetic predisposition, age, and sex (Harber et al., 2017; Lakoski et al., 2011; Lin et al., 2015; Williams et al., 2017). The molecular mechanisms underpinning the cardiometabolic benefits of CRF remain poorly understood (Fiuza‐Luces et al., 2018; Lin et al., 2015). A better understanding of these mechanisms serves an important role in identifying the pathways involved in cardiometabolic health and could facilitate the discovery of new clinical biomarkers, uncover novel therapeutic targets, and help lead the focus of future research (Fiuza‐Luces et al., 2018).

High‐throughput metabolomics offers a promising approach to study the molecular mechanisms underlying CRF (Kelly et al., 2020; Patti et al., 2012). Metabolomics is the comprehensive study of metabolites including carbohydrates, amino acids, lipids and nucleotides (Beger et al., 2016). Previous studies have shown that metabolomic profiles differ between fitness levels (Bork et al., 2025; Morris et al., 2013). Higher levels of physical activity and a higher CRF seem to largely influence lipids and amino acids (Bork et al., 2025; Kelly et al., 2020; Morris et al., 2013). Lower levels of amino acids have been found in participants with higher CRF, whereas lipids have both positive and negative associations with CRF (Bork et al., 2025; Carrard et al., 2022; Morris et al., 2013). Sex‐specific variation in the metabolic associations of CRF has been found; however, sex has not been adequately addressed in most studies (Bork et al., 2025; Carrard et al., 2022; Costanzo et al., 2022). Only a few studies have been conducted in large, population‐based cohorts of healthy individuals, and previous studies have a limited coverage of the metabolome (Carrard et al., 2022). Midlife is a particularly relevant period to study, as it marks the onset of accelerated cardiometabolic risk and represents a key window to study mechanisms relevant for CVD prevention (Rodgers et al., 2019). The present study was conducted within the Prospective investigation of Obesity, Energy and Metabolism (POEM) cohort, which was originally designed to investigate the relationships between obesity and CVD in a population of 50‐year‐old individuals. The current study represents a secondary analysis of this cohort. We aimed to examine the associations between CRF, as assessed by a bicycle exercise test (VO_2_peak), and 790 plasma metabolites in 225 females and 225 males. Furthermore, we applied machine learning methods to explore the relative importance of individual metabolites and metabolite classes in relation to CRF and their added explanatory value beyond traditional CVD risk factors. We hypothesized that metabolites associated with CRF may represent potential biological drivers of cardiometabolic health.

## 2 MATERIALS AND METHODS

### 2.1 Population

This cross‐sectional, population‐based association study of the Prospective investigation of Obesity, Energy and Metabolism (POEM) cohort was conducted in Uppsala, Sweden between 2010 and 2016. Inhabitants of Uppsala city were randomly invited to the study 1 month after their 50th birthday. The participation rate was 26% and participants were included if they self‐reported being healthy and physically able to carry out a bicycle exercise test. A total of 502 participants (249 females, 253 males) were included in the study; the study has been described in detail previously (Lind, 2019). A random sample of 454 participants performed a bicycle exercise test, of whom 450 participants had complete data on CRF and metabolomics, and thus comprise the present study sample. The Prospective investigation of Obesity, Energy and Metabolism (POEM) study was approved by the Ethical Board at Uppsala University (Dnr 2009/057). All study participants provided written informed consent. The study was conducted in accordance with standards of ethics outlined in the Declaration of Helsinki.

### 2.2 Cardiorespiratory fitness

A maximal exercise test with gas exchange (VO_2_peak) was performed using a bicycle ergometer (Jaeger Oxygen Pro, Vyaire Medical, USA). The workload started at 30 W for females and 50 W for males and was increased by 10 W/min until exhaustion. The same cycle ergometer and metabolic cart were used for every participant. None of the participants showed any cardiac, pulmonary, or neuromuscular disease that would restrict the work performance. The VO_2_peak was normalized for lean mass (dual‐energy X‐ray absorptiometry (DXA)) as skeletal muscle is the main site of oxygen consumption during exercise and normalizing to lean mass reduces the influence of body fat, which is strongly related to plasma metabolites.

### 2.3 Covariates

The following covariates were included in the analyses: lab sequence number, education (<10, 10–12 or >12 years), smoking (yes/no), fat mass (%), LDL‐cholesterol, fasting glucose (mmol/L), systolic and diastolic blood pressure (mmHg), and diabetes diagnosis (yes/no). Covariates were collected by a questionnaire about lifestyle, history of diseases, medication, and smoking habits. Fasting plasma samples were obtained and blood pressure was measured according to standard methods. Total and regional body fat and lean mass were estimated using DXA (Lunar Prodigy, GE Healthcare), with all scans performed by the same experienced clinician to minimize potential operator bias.

### 2.4 Metabolomics

Overnight fasting plasma samples for metabolomics analysis were stored at −80°C until processed. Untargeted metabolomics was conducted on plasma samples applying the Metabolon platform (Metabolon Inc., USA), described in detail previously (Lind et al., 2021). In brief, metabolomics analyses were performed by integrating data from four complementary methods: two reverse phases (RP)/UPLC‐MS/MS (ultra‐performance liquid chromatography‐mass spectroscopy/MS) methods with positive ion mode electrospray ionization (ESI), one RP/UPLC‐MS/MS method with negative ion mode ESI, and one hydrophilic interaction liquid chromatography (HILIC)/UPLC‐MS/MS method with negative ion mode ESI. The values were normalized and given in arbitrary units. Missing values are the result of falling below the limit of detection rather than a random processing error; therefore, for each metabolite, Metabolon performs imputation by replacing missing values with its observed minimum (performed after batch normalization). 1265 metabolites were measured; only annotated, non‐xenobiotic metabolites with a detection rate over 75% in all samples were used in the analyses comprising 790 metabolites.

### 2.5 Statistical analysis

Multiple linear regression models were fitted with VO_2_peak (continuous) as the independent variable and the relative concentration of individual metabolites (continuous) as dependent variables. Suitable covariates were chosen by constructing a directed acyclic graph (DAG) (Figure S1) (Textor et al., 2016). Continuous variables were tested for normal distribution by histograms and Shapiro‐Wilks test. Fasting glucose levels were not normally distributed and was log‐transformed to approximate normality. Missing data on smoking (3 missing) and education (1 missing) was imputed by the median. CRF, metabolites and continuous covariates were standardized before analyses. Sex‐stratified analyses were conducted for 225 females and 225 males. Model A adjusted for lab sequence number (ordinal), education (categorical 1–3) and smoking (categorical 0/1) as basic technical and socioeconomic adjustment. Model B adjusted additionally for fat mass (continuous), LDL‐cholesterol (continuous), fasting glucose (continuous), systolic and diastolic blood pressure (continuous) and diabetes diagnosis (categorical 0/1), to further assess associations not mediated by traditional CVD risk factors. Age was not included as a covariate, as all participants were examined within 1 month of their 50th birthday. In a sensitivity analysis, regression coefficients from models with and without age were nearly identical (Model A: *r* = 0.9999 in females and 1.000 in males; Model B: *r* = 1.000 in both sexes). Physical activity was not included as a covariate, as it is closely interrelated with CRF and adjustment would risk removing part of the association of interest. Moreover, our aim was to characterize the metabolomic profile of CRF as a whole, including the part attributable to physical activity. In addition, physical activity was only available as a crude self‐reported measure. Bonferroni adjusted *p*‐value for multiple testing (0.05/790 = *p* < 0.000063) was considered statistically significant in Model A. Only metabolites that met statistical significance according to Bonferroni adjustment in Model A were included in multivariable analyses in Model B where nominal *p*‐value was considered significant (*p* < 0.05). Model fit was assessed by adjusted *R* ^2^ values. Likelihood ratio tests were used to evaluate whether inclusion of significant metabolites significantly improved model fit compared to covariate‐only model. Interaction analysis by sex was performed in the 47 metabolites (27 in females, 24 in males, with 4 identified in both) that were significant in Model A in the sex‐stratified analyses where nominal *p*‐value was considered significant (*p* < 0.05). Directional consistency was assessed for the 47 metabolites that were significant in Model A in the sex‐stratified analyses.

Additionally, a supervised machine learning algorithm random forest was applied (Breiman 2001 Random Forests). Random forest is a tree‐based method and was selected for its strength in capturing non‐linear patterns, managing high‐dimensional data, and reducing overfitting by combining predictions from multiple decision trees to improve predictive performance. In random forest, each tree is trained on a subset sample of the original dataset, with a random subset of variables considered as splitting variables at each node. The final prediction is derived by aggregating the outputs of all individual trees, resulting in enhanced robustness and accuracy. An 80:20 train‐test split was applied, where 80% of the dataset was allocated for training and the remaining 20% for testing. Hyperparameters (number of variables for splitting, number of bootstrapped trees, tree depth) were tuned using tenfold cross‐validation to optimize predictive accuracy and reduce overfitting. Model performance was assessed (in test data) by adjusted *R* ^2^ values. Uniquely to tree‐based machine learning algorithms, variable importance can be calculated. Variable importance measures were extracted to identify the metabolites that were the strongest predictors of VO_2_peak. Additionally, metabolite class (lipids, amino acids, cofactors and vitamins, peptides, carbohydrates, nucleotides, energy and partially characterized molecules) importance was calculated by summing up the individual metabolite importance. Statistical software Stata 17 (StataCorp. 2021. *Stata Statistical Software: Release 17*. College Station, TX: StataCorp LLC.) was used for all analyses. For machine learning analyses r_ml_stata_cv and pyforest2 commands were used.

## 3 RESULTS

Baseline characteristics are presented in Table 1. VO_2_peak normalized to lean mass was higher in females than in males, likely because VO_2_peak increases less than proportionally with lean mass, which gives higher values to individuals with lower lean mass (Nevill et al., 1992; Tanner, 1949). Females and males differed most drastically in fat mass where females had mean fat mass 33.5% and males 22.2%. VO_2_peak was associated with 27 metabolites in females and 24 in males in multivariable linear regression models adjusted for lab sequence number, education and smoking after Bonferroni correction (Tables 2A and 2B; Figure 1). Out of these, 4 metabolites (hydroxy‐CMPF, 4‐hydroxyglutamate, mannose and metabolonic lactone sulfate) were directionally consistent and statistically significant in both females and males, with hydroxy‐CMPF showing a positive association and the remaining metabolites demonstrating inverse associations with VO_2_peak. After additional adjustment for fat mass, LDL‐cholesterol, fasting glucose, diastolic and systolic blood pressure and diabetes diagnosis, 24 and 16 metabolites in females and males respectively remained significantly associated with VO_2_peak (*p* < 0.05) (Tables 2A and 2B). Of the 27 metabolites significantly related to females only and the 24 being related to males only, 10 metabolites showed a significant interaction term between sex and the metabolite (Tables 2A and 2B; Figure 1; Table S1). In females, the 27 metabolites identified as significant in multivariable linear regression Model A together explained 52% of the variance in VO_2_peak (adjusted *R* ^2^). Covariates alone accounted for 26%, inclusion of the 27 metabolites increased explained variance to 53% and significantly improved model fit (likelihood ratio test *p* < 0.001). In males, the 24 significant metabolites explained 34% of variance in VO_2_peak. Covariates explained 27% of the variance, with inclusion of the 24 metabolites increased explained variance to 37% and significantly improved model fit (likelihood ratio test *p* < 0.001) (Table 3). In Random Forest model variable importance rankings of covariates and all 790 metabolites, 9 of the top 15 metabolites were also significant in the multivariate linear regression Model A among females. In males, 8 of the top 15 metabolites identified by the Random Forest model variable importance ranking were likewise significant in multivariate linear regression Model A (Tables 2A and 2B). Most of the highest‐ranking metabolites were either lipids or amino acids. Random forest models including covariates alone explained 35% and 9% of the variance in VO_2_peak in females and males, respectively. Inclusion of the 27 and 24 metabolites identified as significant in multivariable linear regression Model A increased the explained variance to 48% in females and 19% in males (Table 3).

**TABLE 1:** Basic characteristics of study population stratified by sex.

	Females	Males	*p*
	*N* = 225	*N* = 225	
Age (years)	50.2 (0.3)	50.3 (0.1)	0.57
Cardiorespiratory fitness (mL/kg lean mass/min)	50.0 (11.9)	43.7 (8.8)	<0.001
Education			0.026
<10 years	8 (3.6%)	22 (9.8%)	
10–12 years	97 (43.3%)	97 (43.1%)	
>12 years	119 (53.1%)	106 (47.1%)	
Current smoker	25 (11.1%)	12 (5.4%)	0.029
BMI (body mass index) (kg/m^2^)	25.9 (4.8)	26.7 (3.5)	0.046
Fat mass (%)	33.5 (7.0)	22.2 (5.5)	<0.001
Fasting glucose (mmol/L)	5.0 (0.5)	5.1 (1.3)	0.18
LDL‐cholesterol (mmol/L)	3.3 (0.8)	3.6 (0.9)	<0.001
Systolic blood pressure (mmHg)	120 (17)	124 (14)	0.010
Diastolic blood pressure (mmHg)	77 (10)	80 (9)	<0.001
Diabetes diagnosis	5 (2.2%)	5 (2.2%)	1.00

*Note*: Continous variables given as mean (SD) and categorical variables as *n* (%). *p*‐values for categorical variables are from chi‐square tests of the overall distribution between females and males.

**TABLE 2A:** Multivariable regression analyses in females.

Chemical name	Metabolite class	Model A			Model B		
		*β*	(95% CI)	*p*	*β*	(95% CI)	*p*
Imidazole lactate^c^	Amino Acid	0.39	(0.27, 0.52)	3.3e‐09	0.40	(0.25, 0.54)	1.1e‐07^a^
Carotene diol (1)	Cofactors and Vitamins	0.39	(0.27, 0.52)	6.4e‐09	0.27	(0.14, 0.41)	1.3e‐04^a^
Metabolonic lactone sulfate^b^	Partially Characterized Molecules	−0.38	(−0.51, −0.25)	2.2e‐08	−0.17	(−0.30, −0.04)	1.2e‐02^a^
Carotene diol (2)^c^	Cofactors and Vitamins	0.37	(0.24, 0.50)	4.3e‐08	0.25	(0.10, 0.39)	7.2e‐04^a^
1‐cerotoyl‐GPC (26:0)^c^	Lipid	0.36	(0.23, 0.49)	1.4e‐07	0.22	(0.08, 0.36)	2.6e‐03^a^
Hydroxy‐CMPF^b^, ^c^	Lipid	0.34	(0.21, 0.46)	2.7e‐07	0.22	(0.09, 0.36)	1.7e‐03^a^
1‐(1‐enyl‐palmitoyl)‐2‐linoleoyl‐GPC (P‐16:0/18:2)^c^	Lipid	0.35	(0.22, 0.48)	3.1e‐07	0.18	(0.04, 0.32)	1.0e‐02^a^
Branched chain 14:0 dicarboxylic acid	Lipid	0.34	(0.21, 0.47)	4.3e‐07	0.28	(0.13, 0.42)	2.2e‐04^a^
**1‐lignoceroyl‐GPC (24:0)** ^c^	Lipid	0.33	(0.20, 0.46)	1.2e‐06	0.16	(0.02, 0.29)	2.1e‐02^a^
Mannose^b^	Carbohydrate	−0.33	(−0.46, −0.20)	1.4e‐06	−0.14	(−0.27, −0.01)	3.9e‐02^a^
Hydroxyasparagine	Amino Acid	−0.31	(−0.43, −0.18)	3.3e‐06	−0.21	(−0.35, −0.07)	4.1e‐03^a^
Beta‐cryptoxanthin	Cofactors and Vitamins	0.30	(0.17, 0.43)	1.0e‐05	0.22	(0.08, 0.37)	2.9e‐03^a^
**arachidoylcarnitine (C20)**	Lipid	0.30	(0.17, 0.43)	1.4e‐05	0.13	(−0.001, 0.26)	5.3e‐02
4‐oxo‐retinoic acid^c^	Cofactors and Vitamins	0.28	(0.16, 0.41)	1.6e‐05	0.29	(0.14, 0.43)	1.1e‐04^a^
Pimeloylcarnitine/3‐methyladipoylcarnitine (C7‐DC)	Lipid	0.29	(0.16, 0.42)	1.7e‐05	0.16	(0.02, 0.30)	2.8e‐02^a^
Uracil	Nucleotide	0.30	(0.16, 0.43)	1.7e‐05	0.26	(0.11, 0.41)	8.1e‐04^a^
3‐hydroxy‐2‐ethylpropionate^c^	Amino Acid	0.29	(0.16, 0.41)	1.9e‐05	0.28	(0.14, 0.43)	1.6e‐04^a^
4‐hydroxyglutamate^b^	Amino Acid	−0.28	(−0.40, −0.15)	1.9e‐05	−0.22	(−0.36, −0.08)	2.8e‐03^a^
Cortolone glucuronide (1)	Lipid	−0.29	(−0.42, −0.16)	2.4e‐05	−0.22	(−0.36, −0.08)	2.7e‐03^a^
Quinolinate	Cofactors and Vitamins	−0.27	(−0.40, −0.15)	2.4e‐05	−0.11	(−0.25, 0.03)	1.2e‐01
Octadecanedioate (C18‐DC)	Lipid	0.28	(0.15, 0.42)	3.2e‐05	0.27	(0.12, 0.42)	4.3e‐04^a^
1‐(1‐enyl‐palmitoyl)‐2‐oleoyl‐GPC (P‐16:0/18:1)	Lipid	0.29	(0.15, 0.42)	3.4e‐05	0.16	(0.007, 0.30)	4.0e‐02^a^
Carotene diol (3)	Cofactors and Vitamins	0.28	(0.15, 0.41)	3.7e‐05	0.17	(0.02, 0.31)	2.4e‐02^a^
Glycoursodeoxycholic acid sulfate (1)^c^	Lipid	−0.29	(−0.42, −0.15)	4.1e‐05	−0.22	(−0.37, −0.07)	4.4e‐03^a^
3‐methyladipate	Lipid	0.27	(0.14, 0.40)	4.4e‐05	0.23	(0.08, 0.37)	2.2e‐03^a^
1‐linoleoyl‐GPC (18:2)	Lipid	0.28	(0.14, 0.41)	5.4e‐05	0.09	(−0.05, 0.23)	2.0e‐01
Sphingomyelin (d18:2/23:0, d18:1/23:1, d17:1/24:1)	Lipid	0.28	(0.14, 0.40)	5.6e‐05	0.26	(0.14, 0.39)	6.3e‐05^a^

*Note*: Beta‐coefficients represent change in standardized metabolite levels per 1‐unit increase in CRF. Model A: adjusted for lab sequence number, education, smoking. Model B: adjusted additionally for fat mass, LDL‐cholesterol, fasting glucose, systolic and diastolic blood pressure and diabetes diagnosis. Bold indicates significant sex interaction (*p* < 0.05/47).

^a^ Significant in Model B.

^b^ Significant in both females and males.

^c^ In top 15 in random forest variable importance.

**TABLE 2B:** Multivariable regression analyses in males.

Chemical name	Metabolite class	Model A			Model B		
		*β*	(95% CI)	*p*	*β*	(95% CI)	*p*
Gamma‐glutamylisoleucine^c^	Peptide	−0.39	(−0.51, −0.26)	5.8e‐09	−0.23	(−0.37, −0.09)	1.3e‐03^a^
Hydroxy‐CMPF^b^, ^c^	Lipid	0.33	(0.20, 0.46)	5.2e‐07	0.24	(0.10, 0.38)	1.2e‐03^a^
**Sphingomyelin (d18:0/18:0, d19:0/17:0)**	Lipid	−0.33	(−0.46, −0.20)	1.2e‐06	−0.08	(−0.21, 0.04)	1.8e‐01
**Behenoyl dihydrosphingomyelin (d18:0/22:0)**	Lipid	−0.32	(−0.45, −0.19)	3.0e‐06	−0.09	(−0.22, 0.03)	1.4e‐01
**N‐stearoyl‐sphinganine (d18:0/18:0)**	Lipid	−0.30	(−0.42, −0.17)	3.2e‐06	−0.07	(−0.20, 0.05)	2.6e‐01
**Gamma‐tocopherol/beta‐tocopherol** ^c^	Cofactors and Vitamins	−0.31	(−0.44, −0.18)	4.3e‐06	−0.23	(−0.37, −0.08)	3.1e‐03^a^
4‐hydroxyglutamate^b^, ^c^	Amino Acid	−0.30	(−0.42, −0.17)	4.4e‐06	−0.14	(−0.28, −0.01)	4.3e‐02^a^
Gamma‐glutamylvaline	Peptide	−0.31	(−0.44, −0.18)	4.4e‐06	−0.19	(−0.33, −0.05)	8.6e‐03^a^
1‐linoleoyl‐GPG (18:2)	Lipid	−0.30	(−0.42, −0.17)	5.8e‐06	−0.24	(−0.38, −0.09)	1.3e‐03^a^
Cis‐3,4‐methyleneheptanoylcarnitine^c^	Lipid	−0.30	(−0.43, −0.17)	1.2e‐05	−0.23	(−0.38, −0.08)	3.4e‐03^a^
**Sphingomyelin (d18:0/20:0, d16:0/22:0)** ^c^	Lipid	−0.30	(−0.43, −0.17)	1.3e‐05	−0.07	(−0.20, 0.05)	2.4e‐01
Gamma‐glutamylleucine	Peptide	−0.30	(−0.43, −0.17)	1.3e‐05	−0.18	(−0.32, −0.03)	1.8e‐02^a^
**N‐stearoyl‐sphingosine (d18:1/18:0)**	Lipid	−0.29	(−0.42, −0.16)	1.7e‐05	−0.13	(−0.26, 0.01)	5.9e‐02
Gamma‐glutamylglutamate	Peptide	−0.29	(−0.42, −0.16)	2.0e‐05	−0.12	(−0.26, 0.02)	1.0e‐01
Succinylcarnitine (C4‐DC)	Energy	−0.29	(−0.42, 0.16)	2.5e‐05	−0.21	(−0.36, −0.06)	6.1e‐03^a^
Mannose^b^, ^c^	Carbohydrate	−0.29	(−0.42, −0.16)	2.6e‐05	−0.12	(−0.26, 0.01)	7.8e‐02
2‐O‐methylascorbic acid^c^	Cofactors and Vitamins	−0.28	(−0.41, −0.15)	2.9e‐05	−0.17	(−0.32, −0.03)	2.0e‐02^a^
Metabolonic lactone sulfate^b^, ^c^	Partially Characterized Molecules	−0.28	(−0.41, 0.15)	3.1e‐05	−0.17	(−0.32, −0.03)	2.2e‐02^a^
Tigloylglycine	Amino Acid	0.28	(0.15, 0.41)	3.4e‐05	0.15	(0.01, 0.30)	3.9e‐02^a^
1‐stearoyl‐2‐oleoyl‐GPE (18:0/18:1)	Lipid	−0.28	(−0.41, −0.15)	3.8e‐05	−0.13	(−0.27, 0.02)	9.1e‐02
**Gamma‐glutamyltyrosine**	Peptide	−0.27	(−0.40, −0.14)	4.8e‐05	−0.19	(−0.33, −0.05)	1.0e‐02^a^
1‐palmitoyl‐2‐dihomo‐linolenoyl‐GPC (16:0/20:3n3 or 6)	Lipid	−0.28	(−0.41, −0.14)	4.9e‐05	−0.16	(−0.30, −0.02)	2.2e‐02^a^
**N‐palmitoyl‐sphinganine (d18:0/16:0)**	Lipid	−0.28	(−0.41, −0.14)	4.9e‐05	−0.17	(−0.31, −0.03)	1.7e‐02^a^
Pyruvate	Carbohydrate	−0.27	(−0.40, −0.14)	5.9e‐05	−0.16	(−0.31, −0.01)	3.3e‐02^a^

*Note*: Beta‐coefficients represent change in standardized metabolite levels per 1‐unit increase in CRF. Model A: adjusted for lab sequence number, education, smoking. Model B: adjusted additionally for fat mass, LDL‐cholesterol, fasting glucose, systolic and diastolic blood pressure and diabetes diagnosis. Bold indicates significant sex interaction (*p* < 0.05/47).

^a^ Significant in Model B.

^b^ Significant in both females and males.

^c^ In top 15 in random forest variable importance.

**FIGURE 1:**
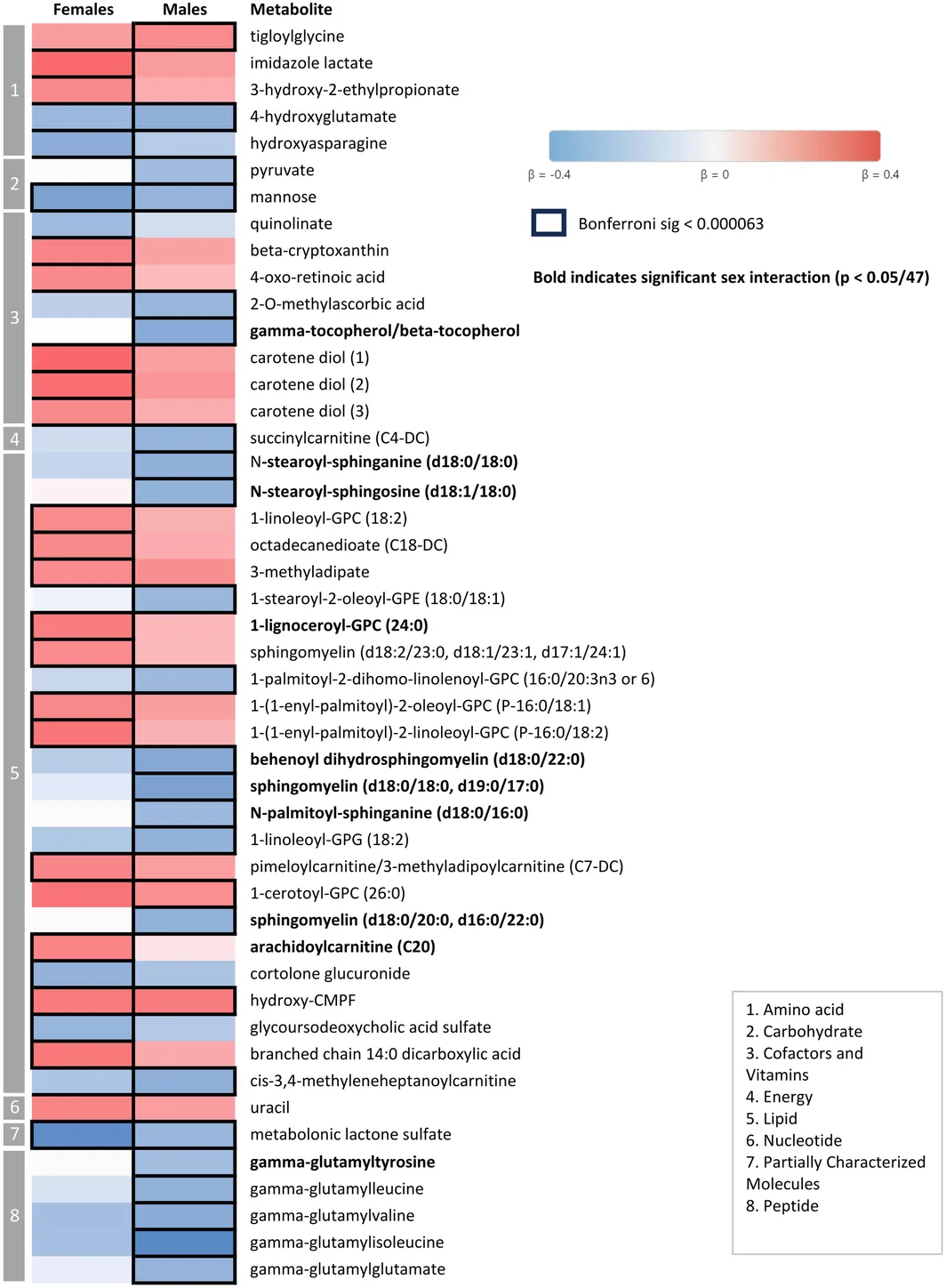
Heatmap. Heatmap of standardized regression coefficients (*β*) for associations between cardiorespiratory fitness (VO_2_peak/lean mass) and plasma metabolites, stratified by sex. Color represents the direction and magnitude of the association, ranging from *β* = −0.4 (blue) to *β* = +0.4 (red). Cells outlined in black indicate Bonferroni‐corrected significance (*p* < 0.000063). Metabolite names in bold indicate a statistically significant sex interaction (*p* < 0.05/47). Numbers correspond to metabolite super‐pathway: 1. Amino acid, 2. Carbohydrate, 3. Cofactors and Vitamins, 4. Energy, 5. Lipid, 6. Nucleotide, 7. Partially Characterized Molecules, 8. Peptide.

**TABLE 3:** Model fit for multivariable linear regression and model performance for random forest regression.

Model	Females	Males
Multivariable linear regression	(likelihood ratio tests)	
Covariates only^b^	0.26	0.27
Significant metabolites only (27 females, 24 males)^c^	0.52	0.34
Covariates + significant metabolites (27 females, 24 males)^b^, ^c^	0.53^a^	0.37^a^

Random forest regression	(adjusted *R* ^2^)	
Covariates only^b^	0.35	0.09
Covariates + all metabolites^b^	0.37	0.13
Covariates + significant metabolites (27 females, 24 males)^b^, ^c^	0.48	0.19

^a^ Significantly improved model fit compared to a covariate‐only model, assessed by likelihood ratio tests (*p* < 0.001).

^b^ Covariates: lab sequence number, education, smoking, fat mass, LDL‐cholesterol, fasting glucose, systolic and diastolic blood pressure, and diabetes diagnosis.

^c^ Significant metabolites: metabolites reaching Bonferroni significance in linear regression Model A (adjusted for lab sequence number, education, and smoking).

## 4 DISCUSSION

### 4.1 Principal findings

We identified associations between CRF and circulating metabolites, with 4 metabolites significant in both females and males. The majority of significant metabolites were sex‐dependent (23 in females, 20 in males). Results indicated both shared and sex‐dependent associations between CRF and metabolites. Most metabolites remained significant after adjustments for traditional CVD risk factors (24 in females, 16 in males), suggesting that metabolic profiling captures biological pathways underlying CRF not reflected in traditional CVD risk factors. Inclusion of significant metabolites significantly improved model fit in regression analyses and model performance in random forest and markedly improved explained variance in both females and males. This suggests metabolites contribute independent predictive value beyond traditional CVD risk factors. Notably, in females, metabolites appeared to be the drivers of CRF variance, whereas in males, covariates and metabolites showed a more additive interplay. The robustness of these findings is further supported by overlap with a machine learning approach, where top‐ranked metabolites from random forest models aligned with linear regression results, and explained variance improved in both models when significant metabolites were included. Results reveal sex‐dependent differences reflected in differences in classes of metabolites and the directionality of associations suggesting sex‐specific metabolic pathways. Interaction analysis identified 10 of the 47 metabolites as showing statistically sex‐dependent associations. Notably, 74% of all 47 associations showed directional consistency across sexes, including the 10 sex‐dependent metabolites, suggesting that sex differences are primarily in the magnitude rather than the direction of associations between CRF and plasma metabolites (Figure 1).

### 4.2 Comparison with literature

To our knowledge, this is the first study to characterize associations between CRF and a comprehensive metabolomics profile in healthy females and males from the general population incorporating a machine learning approach. Our findings both validate and extend previous work. We replicate associations for 6 metabolites (gamma‐glutamylisoleucine, gamma‐glutamylvaline, gamma‐glutamylleucine, succinylcarnitine, gamma‐glutamyltyrosine, pyruvate) previously linked to CRF in a large‐scale study that employed a similar comprehensive sex‐specific approach (Bork et al., 2025). We extend previous findings by additionally adjusting for traditional CVD risk factors. Notably, most metabolites remained significant, suggesting that these associations are not fully explained by established pathways. Metabolites hydroxy‐CMPF and metabolonic lactone sulfate (significant in both sexes) and cortolone glucuronide, carotene diol and pimeloylcarnitine/3‐methyladipoylcarnitine (C7‐DC) (significant in females) have previously been found to be related to all five components of the metabolic syndrome (blood pressure, waist circumference, glucose, HDL‐cholesterol and triglycerides) independent of BMI and sex (Lind et al., 2022). Our results confirm previous findings of sex‐dependent metabolic signatures of CRF, including bidirectional associations between CRF and several lipid‐class metabolites (Bork et al., 2025; Carrard et al., 2022; Kistner et al., 2021). In line with earlier work, amino acids and peptides (chains of amino acids) were predominantly inversely associated with CRF, supporting previous observations of lower amino acid concentrations in individuals with higher CRF (Bork et al., 2025; Carrard et al., 2022; Morris et al., 2013). Lipids and amino acids dominated our top‐ranked metabolites, consistent with previously described influence of CRF on these metabolite classes, although we note that these are the majority classes in general as well (Carrard et al., 2022; Kelly et al., 2020; Kistner et al., 2021). This study addresses limitations of previous work limited by small sample sizes, diseased or athletic populations and less comprehensive metabolomics platforms and a lack of sex‐specific analyses (Carrard et al., 2022; Kelly et al., 2020; Morris et al., 2013).

### 4.3 Potential mechanisms

#### 4.3.1 Metabolites significant in both females and males

Both shared and sex‐dependent metabolic associations have been identified. Metabolites that appear in both sexes may reflect core metabolic responses to a higher CRF. Hydroxy‐CMPF, 4‐hydroxyglutamate, mannose, and metabolonic lactone sulfate were significantly associated in both females and males and span several metabolic domains. *Hydroxy‐CMPF* was positively associated with CRF and is a lipid and a derivative of CMPF (3‐carboxy‐4‐methyl‐5‐propyl‐2‐furanpropionate), a metabolic marker related to fatty acid metabolism and diet (McCullough et al., 2019). CMPF is thought to be beneficial for health as it is associated with inhibition of lipid peroxidation and anti‐inflammatory activity, factors related to CVD (Xu et al., 2017). *4‐Hydroxyglutamate* was negatively associated with CRF and is an amino acid formed from 4‐hydroxyproline inside mitochondria, and the primary source of 4‐hydroxyproline is collagen (dietary and endogenous). Interestingly, higher levels of 4‐hydroxyglutamate have been strongly associated with pre‐term pre‐eclampsia and predictive of gestational diabetes (Sovio et al., 2020; Sovio et al., 2022). Not much is known; it would be interesting for future studies to address whether 4‐hydroxyglutamate is also associated with the risk of CVD, given that females with pre‐eclampsia and gestational diabetes have an increased risk of CVD (Kramer et al., 2019; Smith et al., 2001). *Mannose* was negatively associated with CRF and is a carbohydrate involved in glucose metabolism. Elevated circulating mannose has been associated with more vulnerable plaque and a higher risk of diabetes and cardiometabolic risk (Ferrannini et al., 2022; Sharma et al., 2014). Mannose is emerging as a novel marker for CVD (Ferrannini et al., 2022). *Metabolonic lactone sulfate* was negatively associated with CRF and is a partially characterized metabolite whose chemical structure has not yet been identified. Higher levels have been linked to poor metabolic health with associations with CVD risk factors (Das et al., 2021; Lind et al., 2022).

#### 4.3.2 Sex‐dependent metabolites

Interaction testing identified 10 metabolites with statistically significant sex‐dependent associations with CRF. Of the 10 significant metabolites, 7 were sphingolipids (*sphingomyelin (d18:0/18:0, d19:0/17:0)*, *behenoyl dihydrosphingomyelin (d18:0/22:0)*, *N‐stearoyl‐sphinganine (d18:0/18:0)*, *sphingomyelin (d18:0/20:0, d16:0/22:0)*, *N‐stearoyl‐sphingosine (d18:1/18:0)*, *N‐palmitoyl‐sphinganine (d18:0/16:0)*, *and 1‐lignoceroyl‐GPC (24:0)*). The predominance of sphingolipids suggests that sphingolipid metabolism may be a particularly sex‐sensitive component of the interplay between CRF and metabolites. Most sex‐dependent metabolites (8 of 10) showed stronger inverse associations with CRF in males, including all sphingolipids and *gamma‐glutamyltyrosine*, suggesting that higher CRF in males is particularly associated with lower levels of these metabolites. In contrast, the two metabolites with stronger associations in females, *arachidoylcarnitine (C20)* and *1‐lignoceroyl‐GPC (24:0)*, represent distinct lipid classes, potentially reflecting sex‐dependent differences in fatty acid transport and membrane lipid metabolism with higher CRF.

Also, broader patterns further suggested sex‐dependent associations between CRF and metabolites. First, a notable difference was in the direction of associations, where in females most significant metabolites were higher with higher CRF, suggesting favorable effects of beneficial pathways in fitter females. Whereas, in males most significant metabolites were lower with higher CRF, suggesting reduction of potentially adverse metabolic intermediates with higher CRF. Second, there were differences in dominant metabolic pathways, where in females it was dominated by metabolites involved in lipid metabolism and lipid transportation and antioxidant capacity with higher CRF. In males, the metabolic signature was dominated by lower levels of gamma‐glutamyl peptides with higher CRF, which may reflect improved glutathione metabolism and reduced oxidative stress. Additionally, lower sphingolipids were observed with higher CRF in males, which may reflect a particularly relevant metabolic response to higher CRF with reduced cardiometabolic risk as sphingolipids are strongly linked to insulin resistance and metabolic disease (Meikle & Summers, 2017). Third, differences were observed between the impact of established CVD risk factors. Males lost a greater proportion of significant metabolite associations after adjustment for traditional CVD risk factors (8 vs. 3 in females), suggesting greater overlap between CRF‐associated metabolic pathways and established CVD risk factors in males. This pattern was also reflected in the explained variance: in females, metabolites drove model fit largely independently of covariates, whereas in males, metabolites and CVD risk factors contributed more additively to explaining variance in CRF.

The observed sex differences likely reflect underlying differences in the prevalence and metabolic consequences of traditional CVD risk factors, as well as sex‐specific patterns in diet and lifestyle, all of which may modulate the metabolic associations with CRF (Appelman et al., 2015; Rajendran et al., 2023). Biological differences in body composition further contribute, as females have proportionally more body fat and less skeletal muscle than males at a given BMI (Costa et al., 2025). There are also differences in how females and males process glucose and fatty acids at rest and during exercise where females rely more on lipid oxidation and males on carbohydrates (Costa et al., 2025). These differences are in part hormonally mediated, with estrogens promoting lipid oxidation and testosterone supporting glycolytic capacity (Costa et al., 2025; Mauvais‐Jarvis, 2015).

### 4.4 Clinical implications

While the present findings do not have immediate clinical applicability, the metabolites identified here may serve as candidates for future investigation into the biological pathways linking CRF to cardiovascular health. Notably, several metabolites associated with CRF were not fully captured by traditional CVD risk factors, suggesting they may reflect distinct aspects of metabolic physiology. Understanding these pathways may ultimately inform how fitness‐related metabolic adaptations translate to cardiovascular outcomes. Importantly, the sex‐dependent differences in metabolomic profiles highlighted underscore the need to account for sex in future investigations, an aspect that remains insufficiently addressed in many studies (Carrard et al., 2022; Costanzo et al., 2022).

### 4.5 Strength and limitations

Strengths include objectively measured CRF via maximal exercise testing with gas exchange (VO_2_peak), broad plasma metabolome coverage across 790 metabolites (Metabolon platform), metabolomic profiling by sex, and the application of machine learning to strengthen robustness. Limitations include the cross‐sectional design, which precludes causal interpretation. The study population restricted to 50‐year‐old, predominantly Caucasian individuals from the Uppsala region limits generalizability across age groups and ethnicities. Dietary data, a known confounder of both CRF and the plasma metabolome, was not included in the analyses, as reliable dietary information was unfortunately not available. Finally, menopausal status, which influences metabolism and exercise‐related metabolic responses (Costa et al., 2025; Oosthuyse & Bosch, 2010), was not accounted for in the female‐specific analyses.

## 5 CONCLUSIONS

These findings demonstrate both shared and sex‐dependent metabolic associations with CRF, with inclusion of significant metabolites substantially improving model fit. Results emphasize the importance of considering sex as a biological variable in metabolomic research. This metabolic profiling advances our understanding of the biological pathways linking CRF to cardiometabolic health and identifies candidate metabolites for future biomarker research.

Future studies should explore the causal mechanisms underlying these associations to better understand how shared and sex‐dependent metabolic profiles contribute to cardiometabolic health.

## AUTHOR CONTRIBUTIONS

**Malin Enarsson:** Conceptualization; formal analysis; methodology; project administration; visualization. **Tessa Schillemans:** Formal analysis; methodology. **Hannah L. Brooke:** Methodology. **Lars Lind:** Conceptualization; funding acquisition; methodology. **Johan Ärnlöv:** Conceptualization; formal analysis; funding acquisition; methodology; supervision.

## FUNDING INFORMATION

Malin Enarsson received financial support from the Center for Clinical Research Dalarna (CKFUU‐939626, CKFUU‐936614).

## CONFLICT OF INTEREST STATEMENT

J.Ä. reports payment or honoraria for lectures, presentations, speakers' bureaus, manuscript writing, or educational events from AstraZeneca, Boehringer Ingelheim and Novartis and participation on Advisory Boards with AstraZeneca, Astella, and Boehringer Ingelheim. M.E, T.S, H.L.B and L.L declare no conflicts of interest.

## ETHICS STATEMENT

The Prospective investigation of Obesity, Energy and Metabolism (POEM) study was approved by the Ethical Board at Uppsala University (Dnr 2009/057). The study was conducted in accordance with standards of ethics outlined in the Declaration of Helsinki.

## CONSENT

All study participants provided written informed consent.

## Supporting information


**Table S1:** Interaction analysis by sex on significant metabolites in multivariable regression Model A for women and men.
**Figure S1:** Directed acyclic graph (DAG).

## Data Availability

The data underlying this manuscript originate from the POEM cohort and constitute human data that are considered personal and sensitive under the EU General Data Protection Regulation (GDPR) and Swedish law. According to the ethical approval governing the study and the decisions of the Swedish Ethical Review Authority, this data cannot be made publicly available. However, the data are available to qualified researchers upon reasonable request to the Principal Investigator, Lars Lind (lars.lind@medsci.uu.se), subject to approval by the Swedish Ethical Review Authority and the POEM steering committee. *Code availability statement*: The Stata code used for the statistical analyses in this study is publicly available at Zenodo (https://doi.org/10.5281/zenodo.23259944). The code does not include individual‐level data. The data underlying this manuscript originate from the POEM cohort and constitute human data that are considered personal and sensitive under the EU General Data Protection Regulation (GDPR) and Swedish law. Data is available to qualified researchers upon reasonable request to the Principal Investigator, Lars Lind (lars.lind@medsci.uu.se).
