# Supplementary material for: Cardiorespiratory fitness and plasma metabolites in adults: Sex‐dependent insights from a combined regression and machine learning approach
Source: Physiol Rep. 2026 Nov 13;14(21):e71208. doi: 10.14814/phy2.71208 (PMC13768591; doi:10.14814/phy2.71208)
Supplement: Supplementary file 1 [file PHY2-14-e71208-s001.docx]

| **Supplementary table 1. Interaction analysis by sex on significant metabolites in multivariable regression Model A for women and men.** | | | | |
| --- | --- | --- | --- | --- |
| **Chemical name** | **Metabolite Class** | **Beta-coefficient (95% CI)** | | **p** |
| sphingomyelin (d18:0/20:0, d16:0/22:0) | Lipid | 0.31 | (0.11, 0.51) | .0022* |
| sphingomyelin (d18:0/18:0, d19:0/17:0) | Lipid | 0.30 | (0.10, 0.49) | .0029* |
| arachidoylcarnitine (C20) | Lipid | 0.28 | (0.09, 0.47) | .0046* |
| N-stearoyl-sphingosine (d18:1/18:0) | Lipid | 0.28 | (0.08, 0.47) | .0064* |
| N-palmitoyl-sphinganine (d18:0/16:0) | Lipid | 0.26 | (0.06, 0.45) | .01* |
| gamma-glutamyltyrosine | Peptide | 0.24 | (0.06, 0.42) | .011* |
| gamma-tocopherol/beta-tocopherol | Cofactors and Vitamins | 0.24 | 0.05, 0.44) | .016* |
| N-stearoyl-sphinganine (d18:0/18:0) | Lipid | 0.21 | (0.03, 0.39) | .023* |
| behenoyl dihydrosphingomyelin (d18:0/22:0) | Lipid | 0.22 | (0.03, 0.42) | .024* |
| 1-lignoceroyl-GPC (24:0) | Lipid | 0.20 | (0.00, 0.39) | .048* |
| 1-stearoyl-2-oleoyl-GPE (18:0/18:1) | Lipid | 0.17 | (-0.02, 0.37) | .082 |
| 1-palmitoyl-2-dihomo-linolenoyl-GPC (16:0/20:3n3 or 6) | Lipid | 0.16 | (-0.03, 0.36) | .1 |
| 1-(1-enyl-palmitoyl)-2-linoleoyl-GPC (P-16:0/18:2) | Lipid | 0.15 | (-0.04, 0.34) | .12 |
| pyruvate | Carbohydrate | 0.16 | (-0.04, 0.36) | .12 |
| succinylcarnitine (C4-DC) | Energy | 0.15 | (-0.04, 0.33) | .12 |
| imidazole lactate | Amino Acid | 0.15 | (-0.04, 0.34) | .13 |
| 4-hydroxyglutamate | Amino Acid | 0.13 | (-0.05, 0.32) | .14 |
| gamma-glutamylglutamate | Peptide | 0.13 | (-0.04, 0.31) | .14 |
| 3-hydroxy-2-ethylpropionate | Amino Acid | 0.14 | (-0.05, 0.34) | .15 |
| gamma-glutamylisoleucine | Peptide | 0.11 | (-0.05, 0.28) | .17 |
| 4-oxo-retinoic acid | Cofactors and Vitamins | 0.13 | (-0.06, 0.32) | .18 |
| gamma-glutamylleucine | Peptide | 0.11 | (-0.05, 0.27) | .19 |
| sphingomyelin (d18:2/23:0, d18:1/23:1, d17:1/24:1) | Lipid | 0.12 | (-0.07, 0.31) | .21 |
| carotene diol (1) | Cofactors and Vitamins | 0.12 | (-0.07, 0.31) | .23 |
| 1-linoleoyl-GPG (18:2) | Lipid | 0.10 | (-0.09, 0.28) | .31 |
| cis-3,4-methyleneheptanoylcarnitine | Lipid | 0.09 | (-0.10, 0.28) | .35 |
| metabolonic lactone sulfate | Partially Characterized Molecules | -0.09 | (-0.27, 0.10) | .36 |
| 2-O-methylascorbic acid | Cofactors and Vitamins | 0.09 | (-0.10, 0.28) | .37 |
| branched chain 14:0 dicarboxylic acid | Lipid | 0.09 | (-0.10, 0.28) | .37 |
| gamma-glutamylvaline | Peptide | 0.08 | (-0.09, 0.25) | .38 |
| carotene diol (2) | Cofactors and Vitamins | 0.08 | (-0.11, 0.27) | .41 |
| tigloylglycine | Amino Acid | -0.07 | (-0.26, 0.11) | .42 |
| hydroxy-CMPF | Lipid | -0.07 | (-0.26, 0.11) | .45 |
| octadecanedioate (C18-DC) | Lipid | 0.07 | (-0.12, 0.27) | .46 |
| glycoursodeoxycholic acid sulfate (1) | Lipid | 0.07 | (-0.12, 0.25) | .46 |
| 1-(1-enyl-palmitoyl)-2-oleoyl-GPC (P-16:0/18:1) | Lipid | 0.06 | (-0.13, 0.24) | .53 |
| 3-methyladipate | Lipid | -0.06 | (-0.25, 0.13) | .54 |
| cortolone glucuronide (1) | Lipid | 0.06 | (-0.13, 0.25) | .55 |
| pimeloylcarnitine/3-methyladipoylcarnitine (C7-DC) | Lipid | 0.06 | (-0.14, 0.25) | .56 |
| 1-cerotoyl-GPC (26:0) | Lipid | 0.05 | (-0.15, 0.24) | .64 |
| mannose | Carbohydrate | 0.03 | (-0.16, 0.22) | .76 |
| quinolinate | Cofactors and Vitamins | 0.03 | (-0.17, 0.22) | .8 |
| hydroxyasparagine | Amino Acid | 0.02 | (-0.17, 0.21) | .84 |
| carotene diol (3) | Cofactors and Vitamins | 0.02 | (-0.17, 0.21) | .85 |
| 1-linoleoyl-GPC (18:2) | Lipid | 0.01 | (-0.18, 0.20) | .9 |
| uracil | Nucleotide | -0.01 | (-0.20, 0.18) | .92 |
| beta-cryptoxanthin | Cofactors and Vitamins | 0.01 | (-0.18, 0.20) | .95 |
| * Considered significant p<0.05 |  |  |  |  |

**Supplementary figure 1.** Directed acyclic graph (DAG)

Directed acyclic graph (DAG) illustrating the assumed causal structure between cardiorespiratory fitness and plasma metabolites.
